# Supplementary material for: PRDM6 promotes medulloblastoma by repressing chromatin accessibility and altering gene expression
Source: Sci Rep. 2024 Jul 12;14:16074. doi: 10.1038/s41598-024-66811-6 (PMC11239875; doi:10.1038/s41598-024-66811-6)
Supplement: Supplementary file 1 — Supplementary Information. [file 41598_2024_66811_MOESM1_ESM.pdf]

Supplementary Information for

PRDM6 promotes medulloblastoma by repressing chromatin accessibility and altering gene expression

Christin Schmidt, Sarah Cohen, Brian L. Gudenas, Sarah Husain, Annika Carlson, Samantha Westelman, Linyu Wang, Joanna J. Phillips, Paul A. Northcott, William A. Weiss, Bjoern Schwer

Correspondence:
Bjoern Schwer, UCSF Box 0520, 1450 3rd St., San Francisco, CA 94143; Tel. 415-476-6786; bjoern.schwer@ucsf.edu
William A. Weiss, UCSF Box 0663, 1450 3rd St., San Francisco, CA 94143; Tel. 415-502-1694; william.weiss@ucsf.edu

SUPPLEMENTAL FIGURES AND TABLES

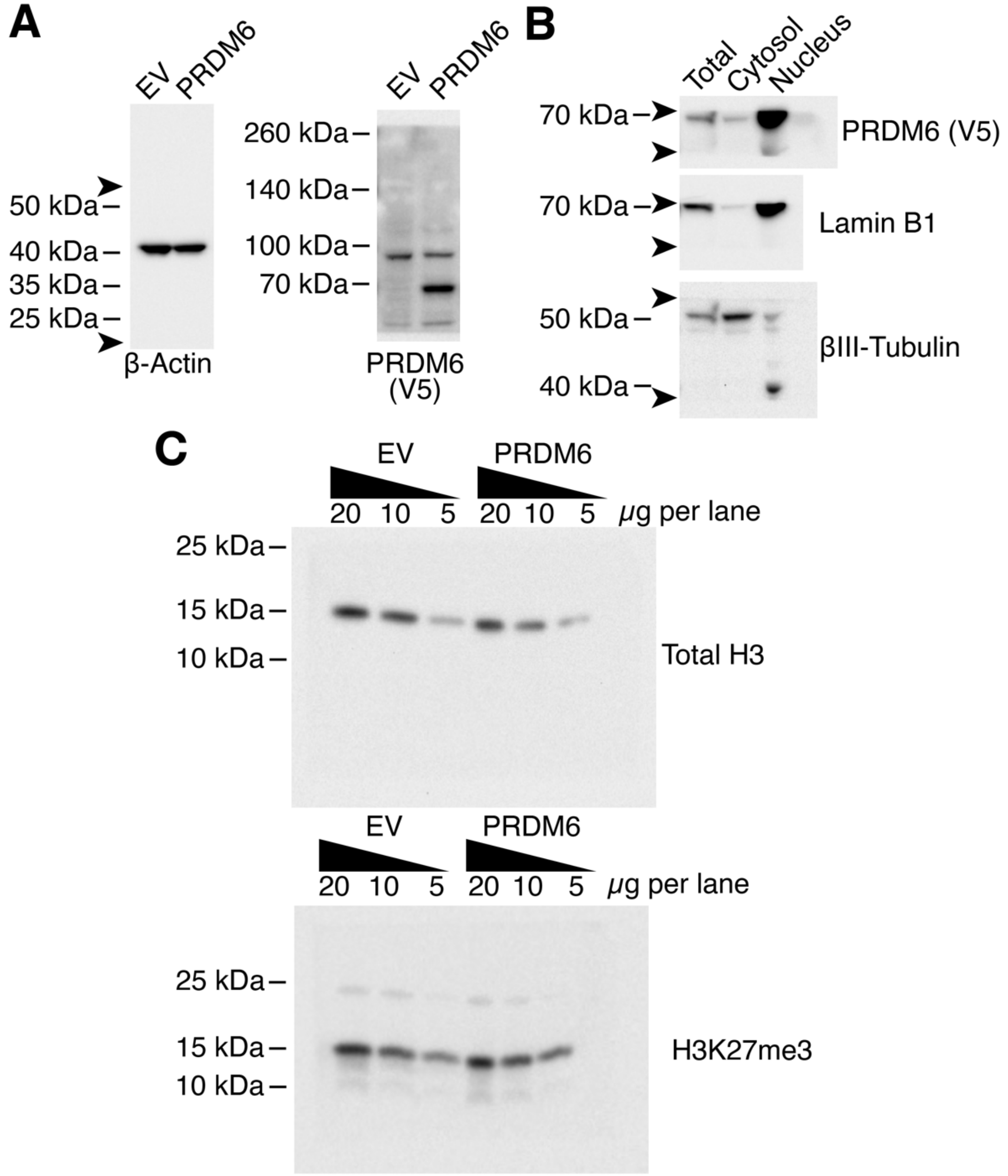

Fig. S1. Original, unprocessed immunoblot images. **(A)** Unprocessed immunoblot images of full-length membranes corresponding to Fig.1C. **(B)** Unprocessed immunoblot images for Fig.1D. Note that these membranes were cut prior to hybridization with the indicated antibodies. The upper and lower edges of the cut membranes are indicated by arrowheads. **(C)** Unprocessed immunoblot images of full-length membranes for Fig. S3A.

A

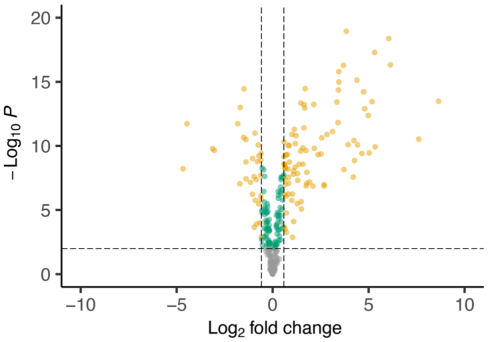

B

Gene Set Name	# Genes in Gene Set	# Genes in Overlap	p-value	FDR q-value
Apoptotic process	1965	61	2.68E-48	2.81E-44
Positive regulation of multicellular organismal process	1684	55	4.05E-44	2.12E-40
Regulation of programmed cell death	1563	53	3.57E-43	1.24E-39
Response to endogenous stimulus	1777	52	4.52E-39	1.18E-35
Locomotion	1335	47	1.32E-38	2.77E-35
Cell motility	1813	50	3.43E-36	5.97E-33
Regulation of cell differentiation	1628	48	6.16E-36	9.2E-33
Negative regulation of programmed cell death	938	40	9.35E-36	1.22E-32
Circulatory system development	1224	42	8.68E-34	1.01E-30
Regulation of locomotion	1142	41	1.02E-33	1.07E-30

Fig. S2. (A) Volcano plot of gene expression of predicted transcription factor target genes in PRDM6 *vs.* EV NES cells. Yellow dots indicate the 122 genes with at least 1.5-fold difference ($P<0.05$) in expression. Most of these (73.8%; 90 genes) are upregulated in PRDM6 NES cells. **(B)** Table of gene set enrichment analysis of the 122 genes upregulated in PRDM6 NES cells and their overlap with all 10,461 Gene Ontology gene sets in the Molecular Signature Database (*Human MSigDB* version *v2023.2.Hs*).

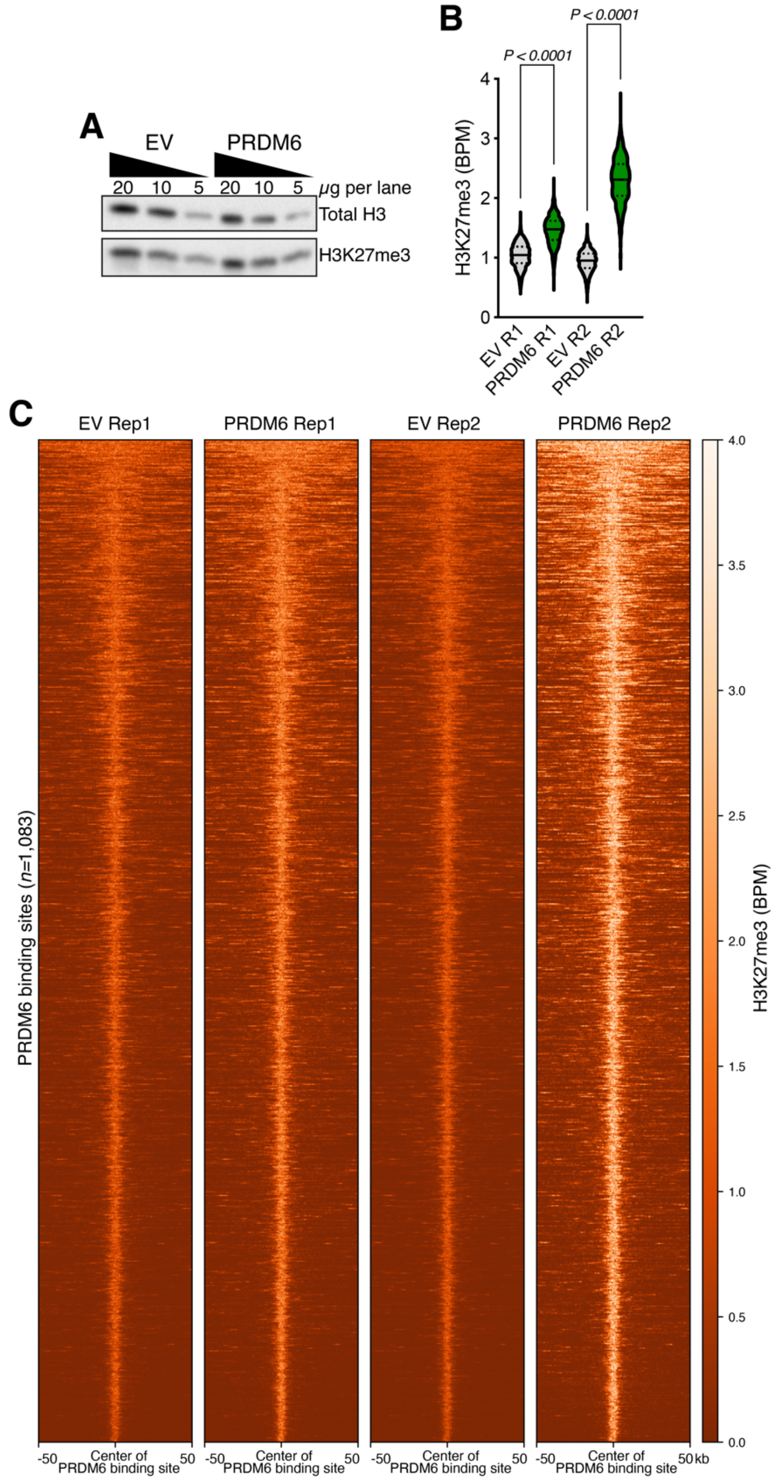

Fig. S3. (A) Global levels of H3K27me3 marks in PRDM6 and EV NES cells. The indicated amounts of acid-extracted histones were analyzed by immunoblotting with H3K27me3-specific antibodies. Blots were stripped and probed for total levels of histone H3. **(B)**

Quantification of H3K27me3 CUT&RUN signal over PRDM6 binding sites in EV and PRDM6 NES cells, respectively. Each violin plot represents BPM-normalized H3K27me3 signal over the 1,083 PRDM6 binding sites identified in NES cells. Solid lines inside the violin plots denote the mean; quartiles are indicated by dotted lines. The *P*-value was determined by ordinary one-way ANOVA. **(C)** Heatmap visualization of BPM-normalized H3K27me3 signal within 50 kb of the center of PRDM6 sites in NES cells.

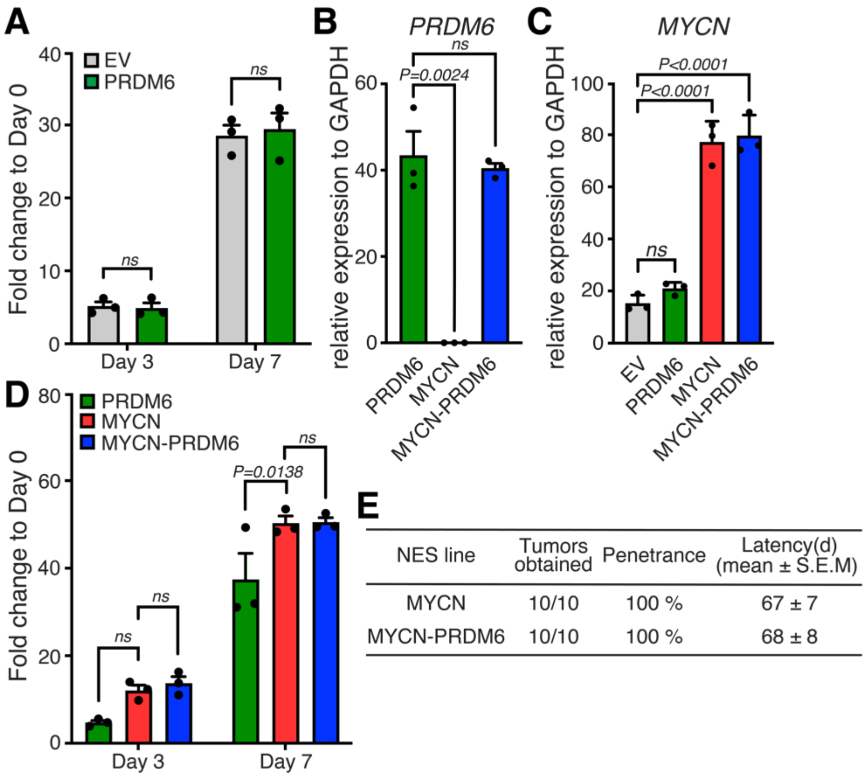

NES line	Tumors obtained	Penetrance	Latency(d) (mean ± S.E.M)
MYCN	10/10	100 %	67 ± 7
MYCN-PRDM6	10/10	100 %	68 ± 8

Fig. S4. (A) *In vitro* proliferation assays of PRDM6 and EV NES cells on days three and seven. Error bars denote SEM. *P*-values were determined by one-way ANOVA with Tukey's *post hoc* correction. **(B)** Quantification of *PRDM6* expression in PRDM6-, MYCN-, or MYCN-PRDM6 NES cells *via* qRT-PCR. Error bars denote SEM. *P*-values were determined by an unpaired, two-tailed *t*-test. **(C)** Quantification of *MYCN* expression, as described in **(B)**. **(D)** *In vitro* proliferation of PRDM6, MYCN and MYCN-PRDM6 NES cells on days three and seven. Error bars denote SEM. *P*-values were determined by one-way ANOVA with Tukey's *post hoc* correction. **(E)** Latency of tumor development after implantation of MYCN- or MYCN-PRDM6 NES cells into the cerebellum of mice. d, days.

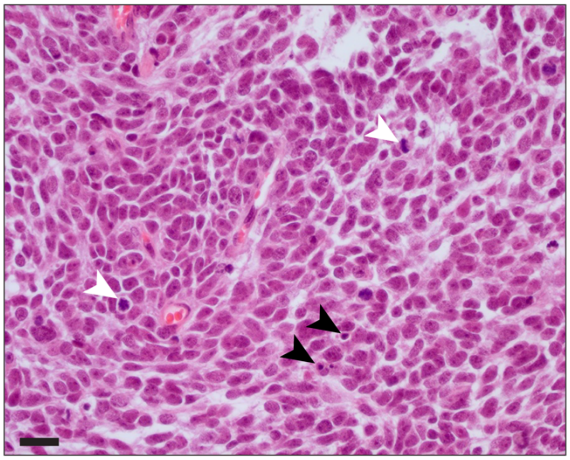

Fig. S5. H&E staining of representative PRDM6 NES-derived tumor tissue. Mitotic figures (white arrowheads) and apoptotic bodies (black arrowheads) are highlighted, respectively. Scale bar, 20 µm.

Table S1. Gene ontology analysis of regions with differential chromatin accessibility. (Excel file).

Table S2. Analysis of genomic regions with differential accessibility for known transcription factor binding motifs. (Excel file).

Table S3. Quantification of occupancy of transcription factor motifs in PRDM6 and EV NES cells. (Excel file).

Table S4. Predicted target genes of transcription factors that show differential binding in PRDM6 *vs.* NES cells.

Table S5. Genes overlapping or located within two kb of a PRDM6 binding site. (Excel file).

Table S6. *Molecular Signatures Database* (MSigDB) analysis of genes overlapping or located within two kb of a PRDM6 binding site. (Excel file).

Table S7. Ranked list of gene pathways used by the random forest classifier. (Excel file).
